# Maleimide-Decorated PEGylated Mucoadhesive Liposomes for Ocular Drug Delivery

**DOI:** 10.1021/acs.langmuir.2c02086

**Published:** 2022-11-03

**Authors:** Roman V. Moiseev, Daulet B. Kaldybekov, Sergey K. Filippov, Aurel Radulescu, Vitaliy V. Khutoryanskiy

**Affiliations:** † Reading School of Pharmacy, University of Reading, Whiteknights, RG6 6DXReading, United Kingdom; ‡ Department of Chemistry and Chemical Technology, Al-Farabi Kazakh National University, 050040Almaty, Kazakhstan; § Forschungszentrum Jülich GmbH, Jülich Centre for Neutron Science (JCNS) at Heinz Maier-Leibnitz Zentrum (MLZ), Lichtenbergstraße 1, 85748Garching, Germany

## Abstract

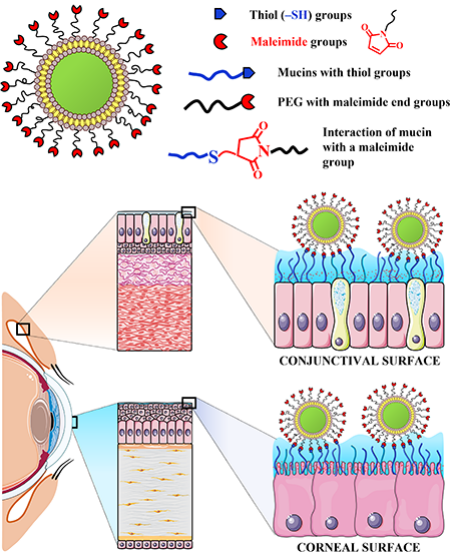

Liposomes are promising spherical vesicles for topical drug delivery to the eye. Several types of vesicles were formulated in this study, including conventional, PEGylated, and maleimide-decorated PEGylated liposomes. The physicochemical characteristics of these liposomes, including their size, zeta potential, ciprofloxacin encapsulation efficiency, loading capacity, and release, were evaluated. The structure of these liposomes was examined using dynamic light scattering, transmission electron microscopy, and small angle neutron scattering. The *ex vivo* corneal and conjunctival retention of these liposomes were examined using the fluorescence flow-through method. Maleimide-decorated liposomes exhibited the best retention performance on bovine conjunctiva compared to other types of liposomes studied. Poor retention of all liposomal formulations was observed on bovine cornea.

## Introduction

The number of people with visual impairment is around 12 million in the United States and over 2.2 billion worldwide.^1^ The human eye is a complex organ with natural barriers for intraocular drug permeation.^2^ Recently, two main approaches have been explored to overcome these limitations, such as enhancing drug penetration into ocular tissues and extending the duration of ocular residence. Both approaches could potentially lead to improved drug bioavailability.

Penetration enhancers are a diverse group of chemical substances facilitating drug permeation through the ocular membranes including cyclodextrins (e.g., hydroxypropyl-β- and γ-cyclodextrins), chelating agents (i.e., ethylenediamine-*N,N*,*N*′,*N*′-tetraacetic acid), crown ethers, surfactants (e.g., Tween-20, polyethylene glycol (PEG) esters), bile acids and bile salts, cell-penetrating peptides, and other amphiphilic molecules (i.e., fatty acids, semifluorinated alkanes, etc.).^3,4^ PEG is a hydrophilic polymer and is widely used in pharmaceutical formulations. PEGylation is an established approach to improve pharmaceutical properties of nanocarriers;^5^ e.g., it increases stability of nanoparticles and improves their mucus penetration properties.^6^ In ocular drug delivery, PEGylation is used to facilitate paracellular transport of drug molecules by inducing a reversible loosening of the epithelial tight junctions.^7^ For example, Eid et al.^8^ demonstrated accelerated permeation through the cornea for PEGylated lipid nanoparticles with a moderate increase in mucoadhesive properties. At the same time, according to Abdul Nasir et al.,^9^ absorption of PEGylated liposomes was slower compared to non-PEGylated nanoparticles, which might be explained by higher resistance to phagocytosis. This might provide a more sustainable drug release. The colloidal stability of PEGylated nanoparticles is thought to be higher with greater molecular weight of PEG due to the more evident steric repulsion.^10^ Balguri et al.^11^ showed that transcorneal permeation of molecules was optimal for PEG with molecular weight ranging from 2000 to 5000 Da. Nevertheless, some researchers have reported the presence of anti-PEG antibodies in patients’ blood, possibly leading to lower drug efficacy and faster blood clearance.^12^ PEGylation remains the gold standard in modifying nanocarriers for drug delivery.^13^

There are several strategies to extend the duration of ocular residence time of the drug formulations, including the use of *in situ* gelling systems divided into three groups according to their phase transition properties: thermosensitive (e.g., Pluronic F127), pH-sensitive (i.e., cellulose acetates and carbomers), and ionic strength sensitive (e.g., gellan gum).^14^ In addition, corneal and conjunctival surfaces are covered with the protective negatively charged glycocalyx.^15^ Hence, mucoadhesive polymers are also commonly used to extend retention time. Depending on the mechanism of action, these polymers can be classified as those favoring ionic interactions (a); those forming strong hydrogen bonds with carboxyl, hydroxyl, and amino groups (b); polymers with high molecular weight (e.g., >100 000 Da in general^16^) and chain flexibility, which are able to interpenetrate into the mucus and forming chain entanglements (c); polymers with surface energy facilitating spreading on the mucus (d).^17^ The mucoadhesive properties of these polymers are mostly based on the mix of several mentioned mechanisms. Thus, ionic interactions (positively charged amino groups of chitosan interact with the negatively charged sialic and sulfonic acids present in the mucin layer), hydrogen bonding, and chain entanglements are the adhesion mechanisms of chitosan, which is considered a gold standard for mucoadhesive polymers.^18^ Chitosan and other cationic and anionic polymers belong to the first generation of mucoadhesive polymers (nonspecific mucoadhesion) and are widely used for enhancing mucoadhesive properties of various formulations.^19^ Bernkop-Schnürch^20^ has developed so-called thiomers (thiolated polymers) as the second generation of mucoadhesive polymers (specific mucoadhesion). Thiol functional groups present in thiomers form covalent disulfide bonds with cysteine residues of mucins produced. They also could facilitate permeation through loosening of tight junctions due to the interaction with thiol ligands of cysteine-bearing membrane receptors and enzymes.^21^

Maleimide is an unsaturated imide widely used for antibody–drug conjugation.^22^ At the same time, it is also known for its mucoadhesive properties due to the formation of carbon–sulfur bonds with thiol groups from the glycocalyx.^23^ It was previously demonstrated by our research group that maleimide-bearing nanogels exhibited improved retention on the conjunctival surface.^24^ In addition, maleimide-functionalized nanoparticles and liposomes have shown good mucoadhesive properties for intravesical drug delivery.^25^ Shtenberg et al.^26^ demonstrated the enhanced mucoadhesive properties of alginate modified with maleimide-terminated PEG drug carriers.

Liposomes are spherical vesicles formed of phospholipid bilayers surrounding an aqueous core. These formulations can contain both hydrophilic and hydrophobic drugs.^27,28^ Liposomal formulations have been demonstrated to deliver drugs to the eye by various researchers over the last few decades.^29^ In addition, modification of the liposomal surface with mucoadhesive polymers (e.g., hyaluronic acid) is a well-known strategy to prolong their retention time on the ocular surface.^30^

In this study, conventional (CL), PEGylated liposomes (with PEG of different molecular weights of 1000 (LPEG1000), 2000 (LPEG2000), 3000 (LPEG3000), and 5000 Da (LPEG5000)) and liposomes decorated with maleimide-terminated PEG (LPEG2000-Mal) were prepared. The size and morphology of these liposomes were characterized using dynamic light scattering (DLS), transmission electron microscopy (TEM), and small-angle neutron scattering (SANS). Encapsulation efficiency (EE%), loading capacity (LC%), and *in vitro* cumulative release of hydrophilic ciprofloxacin·HCl were performed for CL, LPEG2000, and LPEG2000-Mal. Additionally, *in vitro* retention studies on the bovine conjunctival and corneal tissues were conducted for CL, LPEG2000, and LPEG2000-Mal with encapsulated hydrophilic sodium fluorescein (NaFl).

## Materials and Methods

### Materials

Soybean l-α-phosphatidylcholine (PC) was purchased from Alfa Aesar (Heysham, UK). 1,2-Distearoyl-*sn*-glycero-3-phosphoethanolamine-*N*-[methoxy(polyethylene glycol)] (ammonium salt) with different molecular weights of 1000, 2000, 3000, and 5000 Da (DSPE-mPEG1000, DSPE-mPEG2000, DSPE-mPEG3000, and DSPE-mPEG5000) and 1,2-distearoyl-*sn*-glycero-3-phosphoethanolamine-*N*-[maleimide(polyethylene glycol)-2000] (ammonium salt) (DSPE-PEG2000-Mal) were purchased from Avanti Polar Lipids Inc. (Alabaster, USA). Cholesterol (CHO), ciprofloxacin hydrochloride (CF), deuterium oxide (D_2_O), fluorescein isothiocyanate dextran (FITC-dextran, Mw 3000–5000 Da), fluorescein sodium salt, and sodium bicarbonate were purchased from Sigma-Aldrich (Gillingham, UK). Sodium chloride, calcium chloride dihydrate, and phosphate-buffered saline (PBS) tablets (which were used to make 100 mL of 1 × PBS solution in deionized water, pH 7.40) were purchased from Fisher Scientific (Loughborough, UK).

### Preparation of Liposomes

The liposomal formulations containing fixed amounts of l-α-phosphatidylcholine (PC), cholesterol (CHO), and PEGylated lipids at molar ratios of 10:2:0 and 10:2:3 mM (Figure S1 and Table S1 in Supporting Information) were prepared using the thin-film hydration and sonication method.^31^ Briefly, a mixture of PC, CHO, and PEGylated lipids dissolved in chloroform–methanol (2:1, v/v) was transferred into test tubes. The organic solvent was evaporated under a stream of nitrogen, and a thin film of lipid was formed inside the test tubes. The test tubes were vacuum-dried overnight to remove any residual solvent. Then, a solution of 5 mL of PBS was added to the dried lipid films to generate hydrated liposome vesicles, and the tubes were left for 1 h at room temperature. The tubes were vortex-mixed vigorously for 30 min. These dispersions were then sonicated in a sonication bath (FS200b, Decon Laboratories Ltd., UK) for 30 min to reduce the size of the liposomes. Excess lipids were separated from the vesicle formulations by centrifugation of Eppendorf tubes at 13,000 rpm (7558 × g) for 30 min. The supernatants were collected, filtered using 0.22 μm Minisart syringe filters, and stored in a refrigerator prior to characterization. Liposomal formulations encapsulated with 1 mg/mL NaFl (dissolved in PBS) and 1 mg/mL CF (dissolved in 0.9% NaCl) were prepared as above. Solutions of NaFl or CF were used for hydration of the dried lipid films to form liposomes loaded with NaFl or CF.

### Particle Size and Zeta Potential Measurements

The size of liposomes, their polydispersity index (PDI), and zeta potential values were determined using dynamic light scattering (DLS) with a Zetasizer Nano-ZS (Malvern Instruments, UK). Each formulation was diluted 100-fold with Milli-Q ultrapure water. A typical liposome refractive index of 1.45 and absorbance of 0.1 were used in all measurements. Each sample was measured three times at 25 °C, and the mean ± standard deviation values were calculated. The normal resolution analysis model (general purpose algorithm) was selected to obtain intensity-weighted distribution functions over relaxation time. As the next step, the distribution functions over the relaxation time were converted to the distribution functions over hydrodynamic diameters using Stokes–Einstein equation. The ζ-potential values were calculated from the measured values of electrophoretic mobilities using DTS-1070 folded capillary tube cuvettes (Malvern, UK). Polydispersity index (PDI) was taken as an estimate of samples polydispersity. PDI value was calculated as the ratio of second *k*_2_ and first cumulants *k*_1_, PDI = *k*_2_/*k*_1_,^2^ where cumulants *k*_1_ and *k*_2_ are coefficients in the first and second coefficients in the Taylor series of a correlation function *g*_1_(*t*). To obtain the ζ-potential, data was processed using an auto mode analysis model. At least 3 samples were measured and processed using the Smoluchowski model (*F*_κa_ = 1.50) to convert electrophoretic mobility data to ζ-potential.

### Small-Angle Neutron Scattering (SANS) Measurements

Liposome suspensions were prepared as described above using PBS dissolved in deuterium oxide (D_2_O), filtered, and stored in a refrigerator prior to SANS studies. The concentration of nanoparticles used in SANS measurements was ∼5 mg/mL for all liposomes.

SANS experiments were performed at MLZ (Garching, Germany) on a KWS-2 instrument.^32^ Measurements were made on a 3He tube array detector (144 tubes, pixel size 8 mm) using a non-polarized, monochromatic (wavelength λ set by a velocity selector) incident neutron beam collimated with rectangular apertures for three sample-to-detector distances, namely, 2, 8, and 20 m (λ = 0.6 nm). With this setup, the investigated *q*-range was 0.015 nm^–1^ to 4.6 nm^–1^. In all cases, the two-dimensional scattering patterns were isotropic and were azimuthally averaged, resulting in the dependence of the scattered intensity *I*_*s*_(*q*) on the momentum transfer *q* = 4π sin θ/λ, where 2θ is the scattering angle. The curves were corrected for the background scattering from the empty cell and for detector efficiency. Hellma Analytics Suprasil 300 high precision quartz cells of 1 and 2 mm thickness were used in these experiments. SANS experiments were performed in D_2_O solutions. The D_2_O solutions were measured and properly subtracted.

### SANS Data Fitting

The SANS data were fitted by the combination of two models: the model of a lipid bilayer with Gaussian profile of inner and outer layers and the disk form-factor. The models were implemented in SASFit software.^33^ The lipid bilayer is regarded as a planar structure with neutron scattering length for inner hydrophobic and two outer hydrophilic layers (Figure S2).^34^ The disc form-factor is taken to describe the local aspect of liposomes, namely, the thickness. Such an assumption is valid since the membrane thickness is much smaller in comparison with the diameter of liposomes. Thus, liposomes can be regarded as flat structures. The final form-factor is taken in the following way:
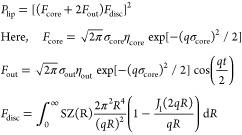
1

The fitting parameters for these models are:

- *σ*_core_: the width of the central Gaussian profile
- *η*_core_: scattering length density contrast of the central Gaussian profile
- *σ*_out_: width of the two outer Gaussian profiles
- *η*_out_: scattering length density contrast of the two outer Gaussian profiles
- *z*: the half of the distance between the centers of the outer Gaussian profiles (*z* = *t*/2)
- *D*: diameter of a planar object (*D* = 2*R*)
- SZ: Schultz-Zimm distribution over radius.

The fitting procedure was started from the fitting of CL where some fitting parameters can be fixed to the values reported in the literature. The values of the fitting parameters obtained for conventional liposomes were taken as initial values for the fitting of the SANS curves of the rest formulations with LPEG1000, LPEG2000, LPEG2000-Mal, LPEG3000, and LPEG5000. The value of χ^2^ was taken as a measure of fitting quality. All values for the diameter obtained from the SANS fitting are below 200 nm, and in the experimental *q* range the lowest *q* = 0.015 nm^–1^ corresponds to the particle size of 418 nm.

### Transmission Electron Microscopy (TEM)

TEM imaging of liposomes was performed using a JEOL 2100 Plus TEM (JEOL, Ltd., Japan) operated at an acceleration voltage of 200 kV. Specimen preparation included pipetting a drop of purified liposomal suspension (∼0.5 mg/mL) onto a parafilm. Then, the drop was covered with the “carbon” side of a glow-discharged holey carbon film-coated 400-mesh copper grid, which was left in contact with each sample for 60 s. A filter paper blotting was used to remove the excess solution. Next, a drop of 1% (w/v) uranyl acetate (UA) solution was placed onto the parafilm, and the “carbon” side with UA was in contact with the grid for 30 s for CL, LPEG2000 and 5 s for the LPEG2000-Mal. The excess stain was dabbed off as described above, and the sample was allowed to dry at room temperature before being characterized with TEM. Previous studies have reported good image quality from this sample preparation method for PLGA–PEG nanoparticles.^25^

### Encapsulation Efficiency (EE%) and Loading Capacity (LC%)

Encapsulation efficiency (EE%) and loading capacity (LC%) for CL, LPEG2000, and LPEG2000-Mal with encapsulated ciprofloxacin hydrochloride (CF) were quantified using Cary 100 UV–vis spectrophotometer (Agilent Technologies, USA) at an absorbance wavelength of 272 nm (Figure S3) and following a modified protocol reported previously within our research group.^25^ Three separate 1 mg/mL solutions of CF were prepared in 0.9% sodium chloride solution. The PBS solution was replaced with 0.9% NaCl, improving the CF’s solubility (pH = 4.84). Then, a calibration curve for CF in 0.9% NaCl solution was produced (Figure 4). Unused Eppendorf tubes were preweighed (*m*_clean Epp_) prior to liposome preparation. After centrifugation, during liposome preparation these Eppendorf tubes with separated excess lipids and residual CF were freeze-dried for at least 12 h. Once completely dried, these tubes were weighed (*m*_lipids+CF+Epp_). Hence, the weight of the mix of excess lipids and residual CF (*m*_lipids+CF in Epp_) was determined using the following equation:
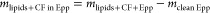
2

Then, the content of each Eppendorf tube was dissolved in MeOH:HCl solution (methanol: hydrochloric acid (1 M) as 70:30 v/v) and transferred into a vial. After 12 h, these solutions (lipids + CF) were 40-fold diluted with 0.9% NaCl solution and analyzed using Cary 100 UV–vis Spectrophotometer. Thus, the concentration of the residual CF (*C*_CF in Epp_) from the *m*_lipids+CF in Epp_ was identified. Next, the weight of excessive lipids (*m*_lipids in Epp_) was calculated using the following equation:
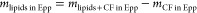
3

Then, 0.5 mL of the lipid nanocarrier dispersion was placed in an ultrafiltration tube with the Amicon Ultra-0.5 Ultracel-3 centrifugal filter unit with a molecular weight cutoff of 3000 Da and centrifuged at 13,000 rpm (7558 × g) for 60 min. The filtrate was discarded. Then, 0.25 mL of 0.9% NaCl was added to wash the retentate before further centrifugation for 40 min. This step was repeated twice. The passed solution was also discarded. The liposomes with CF in the retentate were then disrupted with 0.2 mL of MeOH:HCl solution and centrifuged for 10 min. The solution passed through the filter was collected (from the external tube) and transferred to another marked Eppendorf tube (encapsulated CF). This step was repeated by adding 0.2 mL of MeOH:HCl solution and centrifuged at 4 °C for another 10 min. Then both solutions were collected (from the internal and external tubes) and were transferred into the previous Eppendorf tube (encapsulated CF). Then, this sample was 400-fold diluted with 0.9% NaCl solution and analyzed using the UV–vis spectrophotometer. Hence, the weight of the encapsulated CF (*m*_encaps. CF_) was identified. The encapsulation efficiency (EE%) and loading capacity (LC%) were calculated using the following equations: where *m*_encaps. CF_ is the amount of CF encapsulated in liposomes, *m*_initial CF_ is the initial amount of CF (5 mg for 5 mL), *m*_CF in Epp_ is the residual amount of CF left in Eppendorf tubes during the liposome preparation; *m*_liposomes_ is the total weight of liposomes recovered after centrifugation and *m*_lipids in Epp_ is the weight of excessive lipids left in Eppendorf tubes after the liposome preparation. All the experiments were conducted five times for each sample.
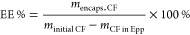
4
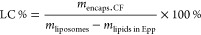
5

### Cumulative Drug Release

*In vitro* cumulative release of CF from CL, LPEG2000, and LPEG2000-Mal was quantified using the UV–vis spectrophotometer at 272 nm. First, 2 mL of each liposomal formulation containing CF was transferred into Pur-A-Lyzer Maxi 3500 dialysis membrane (molecular weight cutoff 3500 Da) and immersed in 30 mL of 0.9% NaCl (pH = 7.40), used as a medium, within 50 mL Falcon tubes. Then, these Falcon tubes were shaken in a water bath at 80 spm for 24 h at 37 °C. At predetermined time intervals (10, 20, and 30 min, and 1, 2, 4, 8, 12, 18, and 24 h), the aliquots (5 mL) were withdrawn from the dialysate and replaced with fresh medium maintaining a constant volume of 30 mL. These aliquots were analyzed without dilution using the UV–vis spectrophotometer (Table S3 in Supporting Information). The cumulative release % for each time point was calculated using the following equation:
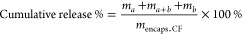
6where *m*_encaps. CF_ is the amount of CF encapsulated in liposomes, *m*_*a*_ is the amount of CF in 5 mL measured in the first time point (10 min), *m*_*a+b*_ is the amount of CF in 5 mL measured in the next time point, *m*_*b*_ is the amount of CF in 30 mL measured at the time point of *b* (for instance, 20 min). All the experiments were conducted five times for each sample. Then, these cumulative release data were analyzed and fitted using the first-order kinetic model with OriginPro 9.8.0.200 software.

### *In Vitro* Retention Studies on Ocular Tissues

The retention properties of CL, LPEG2000, and LPEG2000-Mal with encapsulated 1 mg/mL of NaFl compared to the negative control (1 mg/mL of FITC-dextran solution in deionized water) were evaluated using *ex vivo* bovine cornea and conjunctiva (∼4 × 2.5 cm) tissues following a modified protocol previously developed by our research group.^35^ The intact bovine eyeballs with eyelids were provided by P.C. Turner Abattoirs (Farnborough, UK) straight after the animal slaughter. Tissues were packed and transported in insulated plastic bags. These tissues were delivered to the laboratory within 3 h and were visually assessed in terms of any damage or corneal opacification present. Both corneal and palpebral conjunctival tissues were carefully excised with a scalpel avoiding contact with their surfaces. Prior to experiments, simulated tear fluid (STF) was prepared according to the protocol previously described by Srividya et al.^36^ Thus, 670 mg of sodium chloride, 200 mg of sodium bicarbonate, and 8 mg of calcium chloride dihydrate were dissolved in 100 mL of deionized water at room temperature. The pH of the STF solution was adjusted to 7.40 due to the natural tear pH being around a neutral value.^37^ STF was kept at 37 °C throughout experimentation using a water bath. Freshly dissected tissues were mounted on the glass slides with the testing surfaces facing upward and were stored at 4 °C. Prior to experiments, these slides were placed in an incubator for 1 min at 37 °C. Next, 50 μL of the tested material was applied on the corneal/conjunctival surface with a subsequent STF irrigation using a syringe pump at a flow rate of 100 μL/min for 30 min in the incubator at 37 °C. The flow rate selection aimed to exceed the normal tear production in human eyes (∼1–2 μL/min).^38^ At each time point, the fluorescence images of corneas/conjunctivas were taken using a Leica MZ10F stereomicroscope (Leica Microsystems, UK) fitted with the GFP filter and Leica DFC3000G digital camera at 1.0× magnification, 23 ms exposure time (gain 1.0×). The acquired images were then analyzed using ImageJ software (version 1.50i, 2016) with the mean fluorescence values measured (after each wash with 0.5 mL STF) with the subsequent fluorescence intensity (%) calculation where zero time point was considered as 100% (Tables S4 and S5 in the Supporting Information). At the same time, the image of each tissue without any test material was acquired before the wash-off experiments to measure the blank tissue’s pixel intensity for data normalization. Later, a distribution histogram of the fluorescence intensity values at different wash time points (0 to 30 min with increments of 5 min) was produced as a function of time with the calculated area under the curve (AUC) using OriginPro software (version 2021; OriginLab Corporation, USA). All retention tests were conducted in triplicate for each sample.

### Statistical Analysis

All the experimental data values are calculated as mean ± standard deviation. To estimate the statistical significance a one-way analysis of variance (ANOVA) and two-tailed Student’s *t* test were used, where *p* < 0.05 considered as significant.

## Results and Discussion

### Physicochemical Characterization

**Figure 1:**
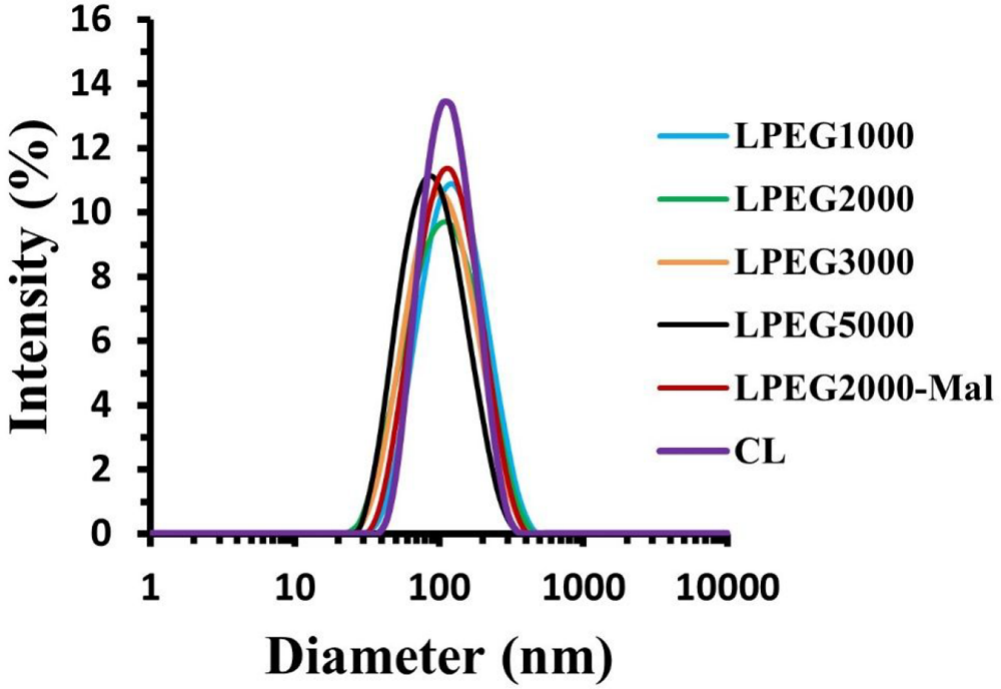
Intensity-weighted size distribution of conventional, PEGylated, and PEG-Mal liposomes as determined by DLS.

For the DLS measurements CL, LPEG1000, LPEG2000, LPEG3000, LPEG5000, and LPEG2000-Mal were prepared using PBS solution and without any drug encapsulated. These data are presented in Table 1 and Figure 1. One-way ANOVA testing demonstrated that there were statistically significant differences between CL and PEGylated liposomes, including LPEG2000-Mal, but no difference was observed between LPEG2000 and LPEG3000 (*p* > 0.05). It is also interesting to note that LPEG1000 are significantly greater in size than CL. This could be related to the presence of PEG corona. LPEG2000-Mal were slightly greater than LPEG2000 with the significant difference (*p* < 0.05) between LPEG2000 and LPEG2000-Mal. This can be explained by the presence of the terminal maleimide groups. On the other hand, LPEG5000 displayed a particle size even smaller than CL. This could be related to a slightly different structural organization of their core. According to the literature, the most optimal size of nanoparticles for uptake by the conjunctiva and cornea is considered to be less <200 nm.^39^ Moreover, the obtained size data demonstrate the presence of small unilamellar vesicles, which are widely used in clinically approved products.^27^ The polydispersity index (PDI) of all types of liposomes was <0.23, indicating a homogeneous liposomal population with a narrow size distribution. The PDI is a measure of the size distribution, and according to the literature, liposome formulation is considered to be heterogeneous if PDI is ≥0.30.^40^ Vesicles showing their zeta potential less than −30 mV are believed to have good colloidal stability of the nanodispersion system and have a reduced number of bilayer membranes due to the electrostatic repulsion between charges of the same polarity.^41^ Additionally, liposomal formulation with ≤ −30 mV would have higher entrapment capacity, because stronger zeta potential contributes to the increase in the unilamellar vesicles.^42^

**Table 1:** Physicochemical Characteristics of Conventional, PEGylated, and PEG-Mal Liposomes Determined by DLS and SANS

	DLS			SANS					
Liposomal formulations	Mean diameter (nm)	PDI	Zeta potential (mV)	*D* (nm)	*σ*_out_ (nm)	*η*_out_, 10^–7^ (Å^–2^)	*σ*_core_ (nm)	*η*_core_, 10^–7^ (Å^–2^)	*z* (nm)
CL	101 ± 1	0.156	–58 ± 1	139	0.54	7.2	13.2	0.3	1.8
LPEG1000	110 ± 1	0.221	–53 ± 1	158	1.05	3.6	9.9	0.2	1.4
LPEG2000	94 ± 1	0.207	–41 ± 1	110	0.59	7.7	4.7	0.8	1.7
LPEG2000-Mal	97 ± 1	0.228	–42 ± 1	124	0.54	6.8	5.6	0.6	1.7
LPEG3000	93 ± 1	0.227	–36 ± 1	102	0.45	9.1	3.1	1.8	1.6
LPEG5000	83 ± 1	0.210	–29 ± 1	86	0.41	11.1	3.7	1.5	1.6

**Figure 2:**
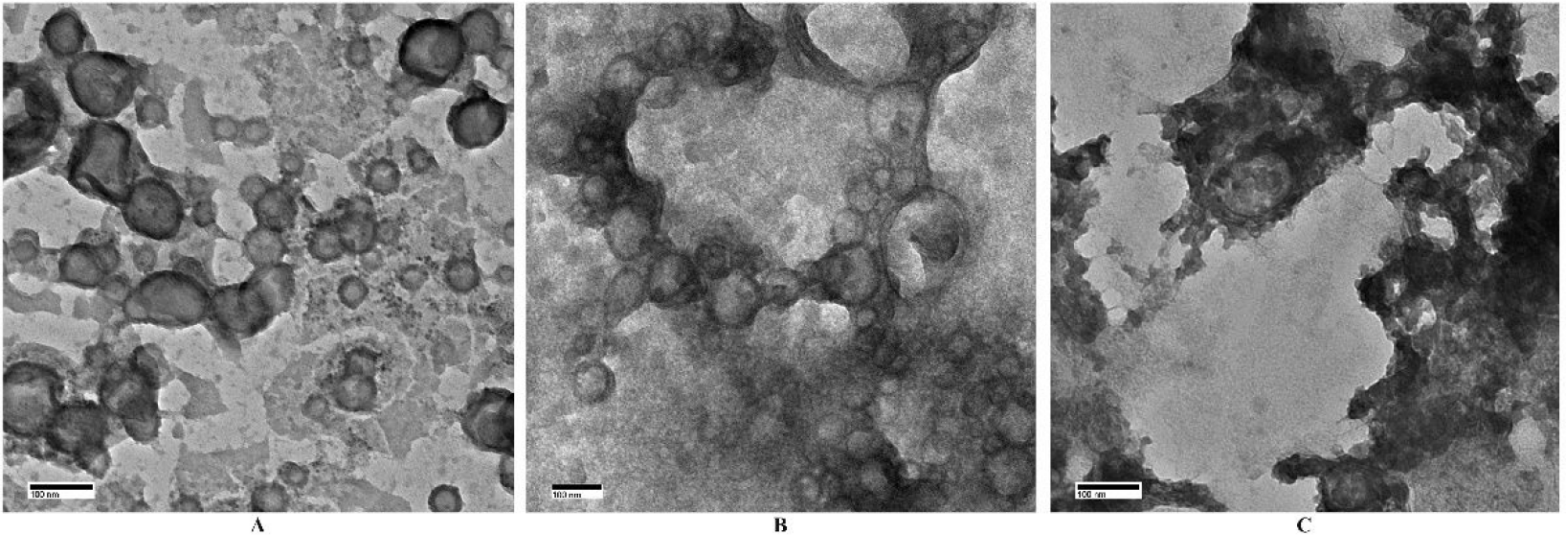
Transmission electron microscopy (TEM) images of CL (a), LPEG2000 (b), and LPEG2000-Mal (c). Scale bars are 100 nm for all images with direct magnification of 25,000× for (a) and (c), and 20,000× for (b).

The negative staining TEM analysis with uranyl acetate was used to confirm the size and morphology of CL, LPEG2000, and LPEG2000-Mal, and the microphotographs are shown in Figure 2. The obtained data demonstrated that homogeneous liposomes were formed with clear spherical morphology, confirming the DLS data (Table 1).

### SANS

**Figure 3:**
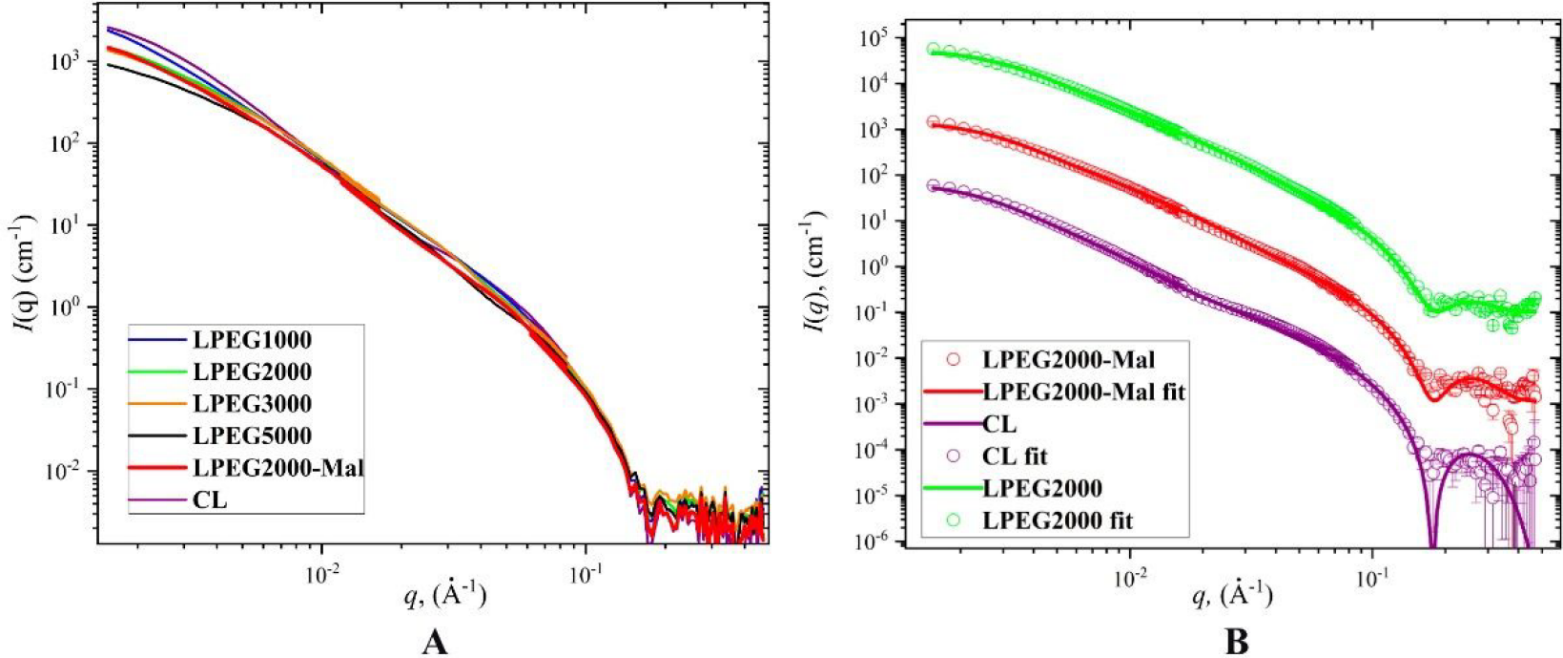
SANS scattering curves. The experimental (A) and selected (B) SANS data with their fits. Please note that the scattering curves are vertically shifted for clarity in part B.

Having obtained the proof of a vesicular structure from TEM experiments, SANS experiments were performed to shed light on the detailed information in the liposome membrane. All SANS curves were successfully fitted by the model described above (Table 1, Figure 3 (A and B)) giving additional confirmation of the vesicular morphology of the nanoparticles. Several features should be noted here. Visual inspection of the scattering curves gives evidence of the lack of the drastic changes in liposome structure with increasing PEG length; liposomes preserve their bilayered structure with incorporation of PEG. Any significant modification of nanoparticle shape such as liposome destruction, vesicle-to-micelle transformation, or strong liposome aggregation would be immediately manifested on a scattering curve.^43^ Second, there is a strong agreement and correlation between the sizes measured by DLS and diameter values obtained from SANS fitting (Table 1). Such an agreement is an additional proof of the good fitting quality. The same as for DLS results, the correlation between the liposomes diameter values and PEG length is visible from the fitting data (Table 1). The Pearson coefficient is −0.86. The values of interlayer distance *z* are barely sensitive to the presence of PEG. The obtained *z* values are in agreement with previously reported data.^34^ The SANS data also provide evidence that the width of the outer hydrophilic layer is nearly constant over the PEG length. However, the decrease of the width of the central Gaussian layer from 13.2 to 3.1 nm with the increasing PEG chain implies that the inner hydrophobic layer is getting a more uniform structure. Comparison between the LPEG2000 and LPEG2000-Mal samples with and without maleimide groups shows that maleimide groups are possibly partially incorporated into a hydrophobic shell making it less uniform than the Mal-free sample.

### Encapsulation Efficiency (EE%) and Loading Capacity (LC%)

The pH of CF solution was measured as 4.84 ± 0.01. Ideally, it is recommended to formulate eye drops with a pH close to the physiological pH of tear fluid in order to reduce discomfort and minimize lacrimation. Still, a human eye can tolerate topical ophthalmic formulations with pH values between 3.5 and 9.^27^ In addition, the pH of the commercially available CF eye drops is ∼4.5.^44^ The entrapment efficiency and loading capacity values of CL, LPEG2000, and LPEG2000-Mal are within the ranges of 27–28% and 1–5%, respectively, without any statistically significant difference between them (Table S2 in Supporting Information). These figures are broadly comparable to the literature sources.^45^ Hosny reported figures of EE% for their liposomes within the liposomal hydrogel as high as 82%, which might be explained with a different liposomal composition including the addition of stearylamine and dicetyl phosphate.^46^ Standard deviations reaching up to 40% of the average figure might be explained by the presence of multilamellar liposomes in the population.

### *In Vitro* Cumulative Release

**Figure 4:**
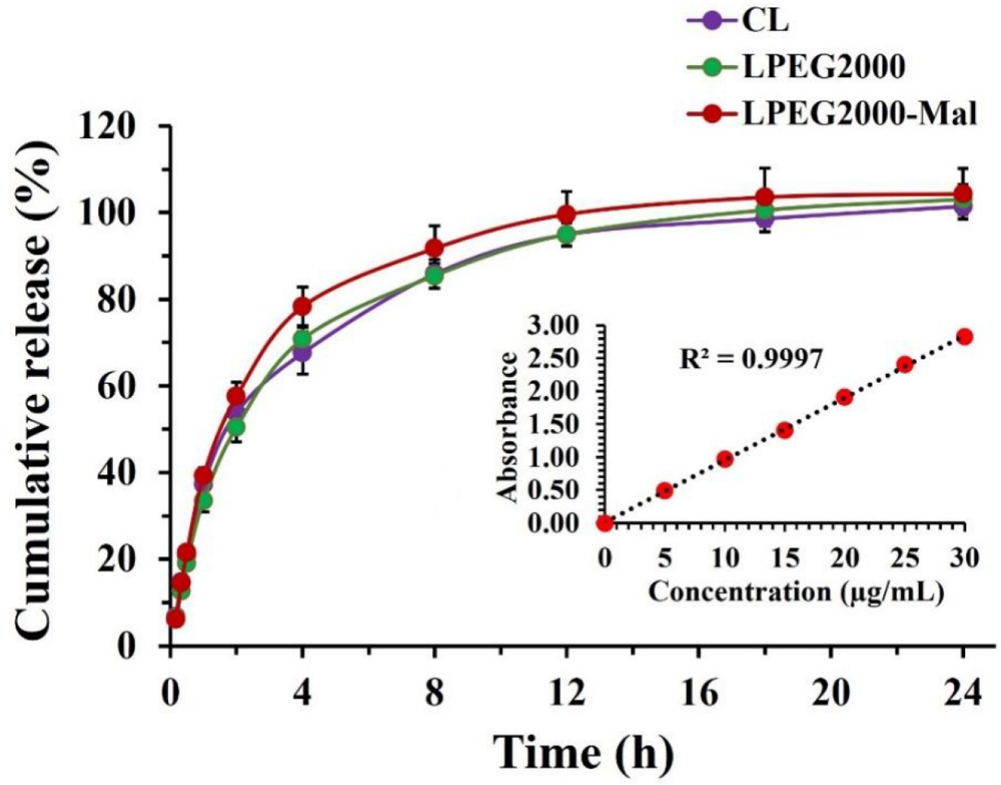
Experimental release profiles of CF from CL, LPEG2000, and LPEG2000-Mal with the inserted calibration curve. Data set is expressed as mean ± standard deviation (*n* = 5).

A dialysis method was used to determine the cumulative release profile of CF from CL, LPEG2000, and LPEG2000-Mal at 37 °C in STF solution. The experimental cumulative release profiles are presented in Figure 4, while numerical values are shown in Table S3 in Supporting Information. All cumulative release curves were successfully fitted using the first-order kinetics model (Figure S4 in the Supporting Information), considered to be a common drug release model for liposomal formulations. Interestingly, the drug release profiles from the CL, LPEG2000, and LPEG2000-Mal were similar, reaching ∼100% by ∼12–18 h without statistically significant difference between them. According to the literature data, the unilamellar liposomes with a hydrodynamic diameter of ∼130 nm exhibit a higher drug release rate compared to the multilamellar liposomes with two or three bilayers and a diameter of ∼250 nm. In general, this difference in release rate results from the number of phospholipid bilayers the encapsulated drug needs to cross.^47^

### *In Vitro* Retention Studies on Ocular Tissues

The corneal epithelium is known to be the major barrier to topical drug delivery to the eye.^48^ On the other hand, stromal permeability is dependent on the molecule radius, providing a strong limitation for lipophilic compounds of small size (radius <10 Å). Interestingly, the endothelium layer is slightly less permeable to small lipophilic molecules than the corneal stroma. Macromolecules, however, cross the endothelium more readily than stroma. According to the literature, the conjunctiva is 8.6 ± 4.4-fold more permeable than the cornea.^49^ Hamalainen et al.^50^ demonstrated greater permeability of a rabbit’s conjunctiva compared to the corneal tissues for the mixture of PEGs with the mean molecular weights of 200, 400, 600, and 1000 Da.

**Figure 5:**
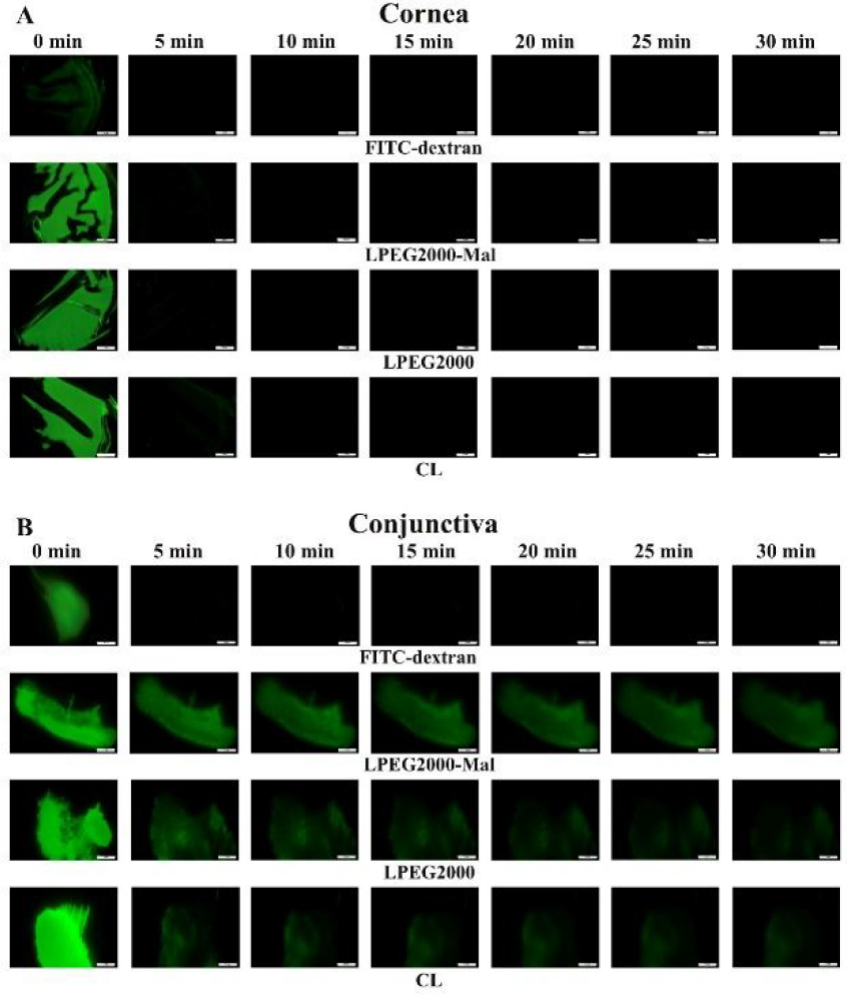
Exemplar images of *ex vivo* bovine corneal (A) and conjunctival (B) tissues with applied FITC-dextran solution, CL, LPEG2000, and LPEG2000-Mal. Scale bars are 5 mm.

**Figure 6:**
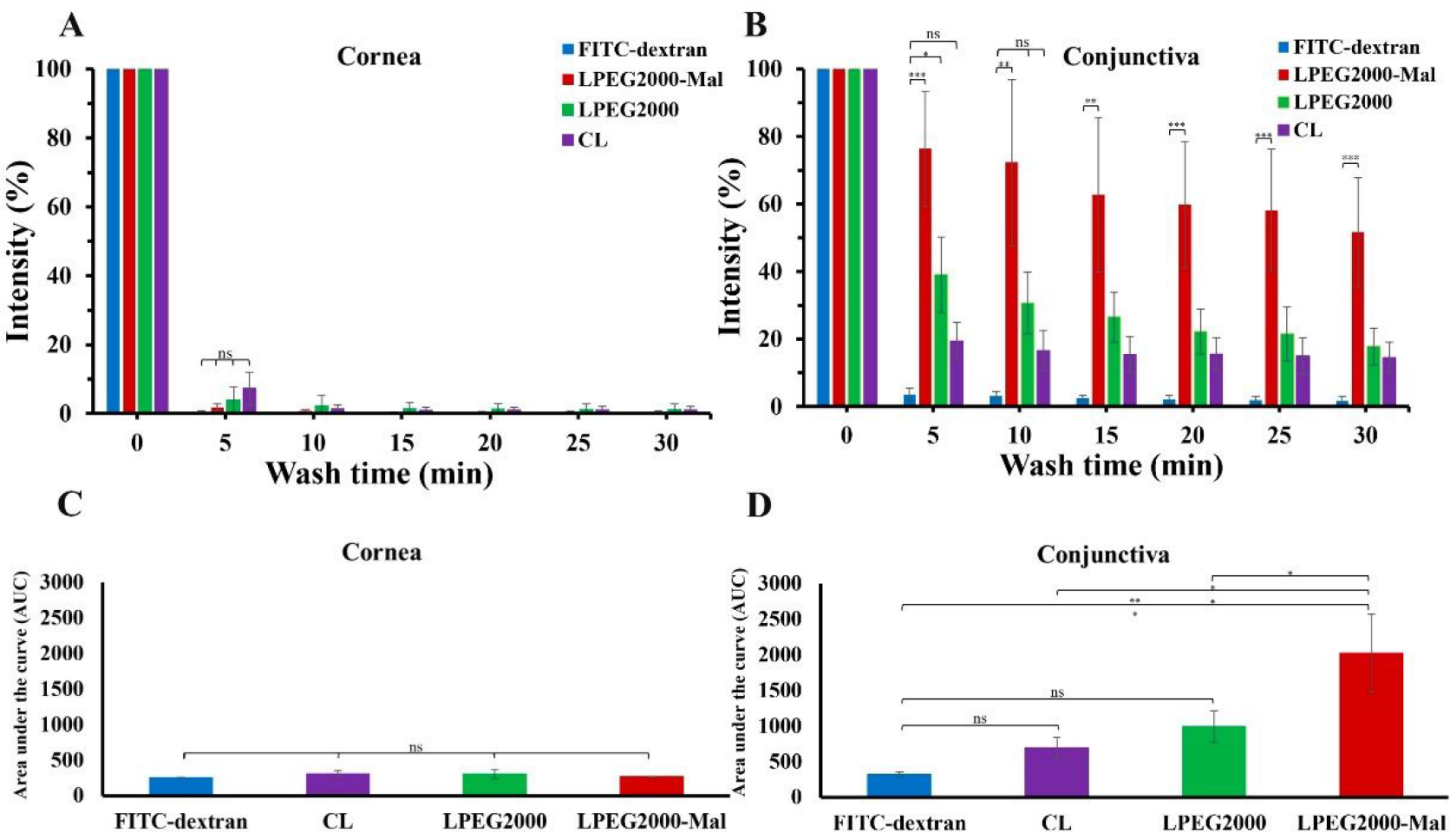
Mucoadhesive properties assessment for CL, LPEG2000, and LPEG2000-Mal in comparison to FITC-dextran in deionized water using a wash-off test for 30 min on *ex vivo* bovine cornea (A) and conjunctiva (B). Area under the retention curve values represent mucoadhesive properties of FITC-dextran solution and liposomes on cornea (C) and conjunctiva (D). The percentage values are expressed as mean ± standard deviation (*n* = 3). Statistically significant differences were represented as *** – *p* < 0.001; ** – *p* < 0.01; * – *p* < 0.05; ns – no significance.

The results generated during the *in vitro* retention experiments supported these data. Thus, CL, LPEG2000, and LPEG2000-Mal with 1 mg/mL NaFl demonstrated very poor retention on *ex vivo* bovine cornea (Figure 5A and Figure 6 (A and C)), and average fluorescence values can be found in Tables S4 in Supporting Information. All three types of liposomes were washed off the corneal surface by the fifth minute. At the same time, no statistically significant difference was detected between these liposomes for area under the curve (AUC) calculations for the total time of the mucoadhesion experiment (0 to 30 min with 5 min increments).

A completely different retention of liposomes is observed for the bovine conjunctival tissues. These results with the exemplar images are presented in Figure 5B and Figure 6B,D. The average fluorescence values and AUC figures are shown in Table S5 in Supporting Information. After the first 5 min of STF washing, significantly greater retention of LPEG2000-Mal was observed in comparison with the negative control (1 mg/mL FITC-dextran solution) and CL (*p* < 0.001 and *p* < 0.01, respectively). There was also a small but significant difference between LPEG2000-Mal and LPEG2000 (*p* < 0.05). CL demonstrated no statistically significant difference with the negative control. Following 10 min of the experiment LPEG2000-Mal were the only formulations that showed a difference compared to FITC-dextran (*p* < 0.01). Surprisingly, there was no difference between LPEG2000 and the negative control, which might be potentially explained by the presence of PEG rather than the combination of PEG and maleimide like in LPEG2000-Mal. The same retention was observed after 15 min. From 20 min onward up to 30 min of the retention test, the difference between LPEG2000-Mal and negative control became even higher, reaching *p* < 0.001. The potential permeation of NaFl through the conjunctiva might explain this higher difference, which results from the prolonged presence of these liposomes on the mucosal surface due to the formation of covalent bonds with thiol groups from the glycocalyx. At the same time, AUC calculations demonstrated a significant difference between LPEG2000-Mal and FITC-dextran, conventional, and LPEG2000 (*p* < 0.001, *p* < 0.01, and *p* < 0.05, respectively). No statistically significant difference was observed across negative control, CL, and LPEG2000.

**Figure 7:**
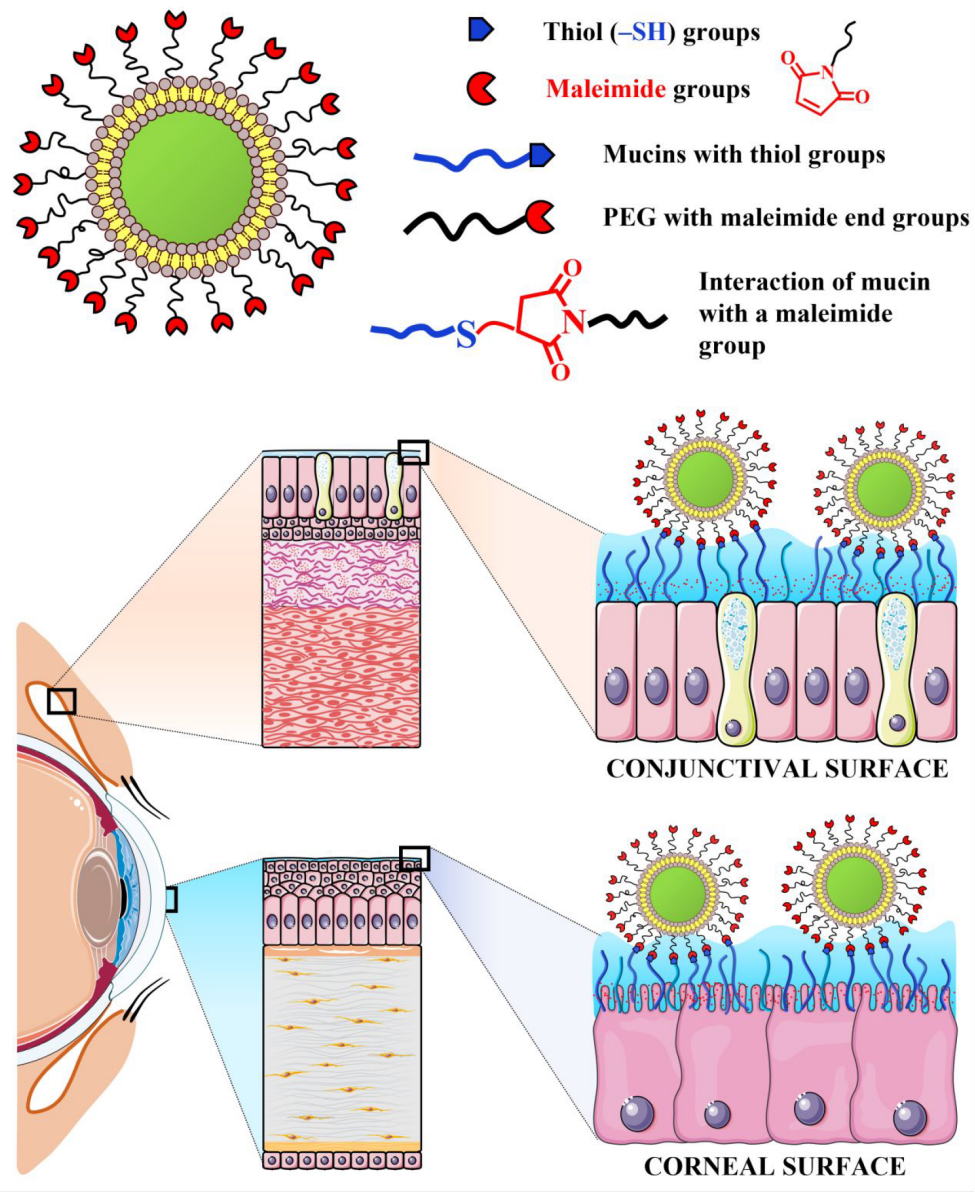
Structure of liposomes functionalized with maleimide groups (LPEG2000-Mal) and their possible reaction with thiol groups present on the cornea and conjunctiva.

The schematic structure of LPEG2000-Mal liposomes and their possible reaction with thiol groups present on the surfaces of the cornea and conjunctiva are shown in Figure 7. There are several possible reasons for better retention of maleimide-decorated liposomes on the conjunctiva compared to the corneal tissues. These include increased permeability of the conjunctiva compared to the cornea^48−50^ and higher density of the goblet cells within the conjunctiva, resulting in a greater amount of mucin produced.^51^ In addition, membrane-bound mucins are produced by both the cornea and conjunctiva, but the conjunctiva also secretes soluble mucins.^52^

## Conclusions

Conventional, PEGylated (with different molecular weights of 1000, 2000, 3000, and 5000 Da), and maleimide-decorated liposomes were formulated in this study. The physicochemical characteristics of these nanoparticles, encapsulation efficiency, loading capacity, ciprofloxacin drug release, and *ex vivo* corneal and conjunctival retention were examined. Very poor retention of all liposomal formulations was observed on the cornea, but substantially better retention of these vesicles was seen on the conjunctiva. The maleimide-decorated liposomes demonstrated the best performance on bovine conjunctiva due to the ability of maleimide groups to form covalent bonds with thiol groups present in mucins. As a result of these studies, liposomes decorated with PEG with terminal maleimide groups are shown to have the potential for improved retention on the conjunctiva in topical drug delivery to the eye.

## Supporting Information Available

Schematic representation of preparation of CL, PEGylated and PEG-Mal liposomes; SLD profile for a lipid bilayer with a Gaussian electron density distribution; composition (%w/v) of lipid nanocarrier formulations; UV–vis spectra with maximum absorbance peak for CF dissolved in 0.9% NaCl solution; encapsulation efficiency (EE%) and loading capacity (LC%); CF cumulative release (%) from CL, LPEG2000, and LPEG2000-Mal; selected release profiles of CF from CL, LPEG2000, and LPEG2000-Mal; Average fluorescence intensity (a.u.) and AUC values for mucoadhesion tests (PDF)
