# Supplementary material for: Maleimide-Decorated PEGylated Mucoadhesive Liposomes for Ocular Drug Delivery
Source: Langmuir. 2022 Nov 3;38(45):13870–9. doi: 10.1021/acs.langmuir.2c02086 (PMC9671038; doi:10.1021/acs.langmuir.2c02086)
Supplement: Supplementary file 1 — la2c02086_si_001.pdf [file la2c02086_si_001.pdf]

Supporting information

Maleimide-Decorated PEGylated Mucoadhesive Liposomes for Ocular Drug Delivery

Roman V. Moiseev[a,1], Daulet B. Kaldybekov[a,b,1], Sergey K. Filippov[a], Aurel Radulescu[c], Vitaliy V. Khutoryanskiy[a,*]

[a] Reading School of Pharmacy, University of Reading, Whiteknights, RG6 6DX Reading, United Kingdom

[b] Department of Chemistry and Chemical Technology, Al-Farabi Kazakh National University, 050040 Almaty, Kazakhstan

c Forschungszentrum Jülich GmbH, Jülich Centre for Neutron Science (JCNS) at Heinz Maier-Leibnitz Zentrum (MLZ), Lichtenbergstraße 1, 85748 Garching, Germany

[1] These authors contributed equally.

*Corresponding author

Postal address: Reading School of Pharmacy, University of Reading, Whiteknights, PO Box 224, RG6 6DX Reading, United Kingdom

E-mail address: v.khutoryanskiy@reading.ac.uk (V.V. Khutoryanskiy)

Phone: +44(0) 118 378 6119

Fax: +44(0) 118 378 4703

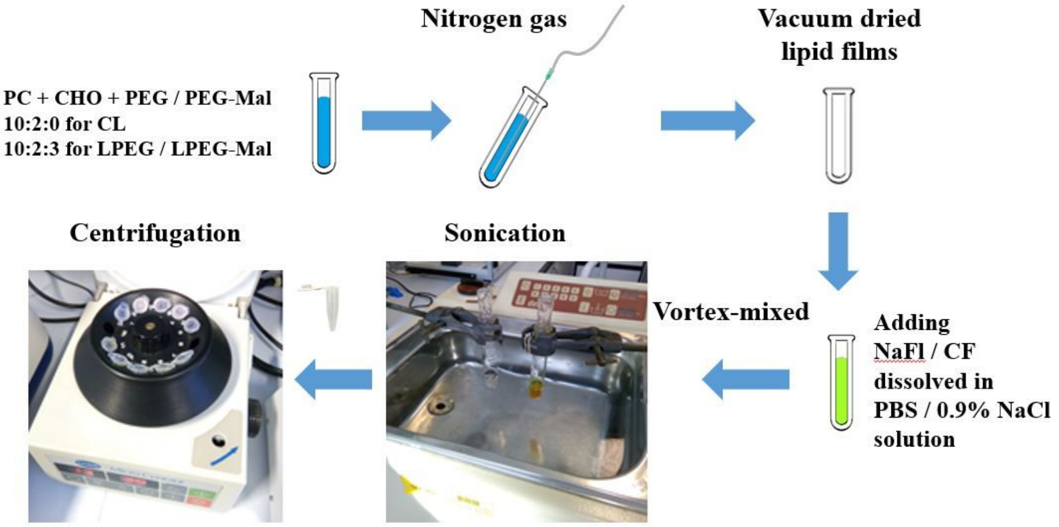

Figure S1. Schematic representation of preparation of CL, PEGylated and PEG-Mal liposomes without drug and with encapsulated fluorescein sodium salt (NaFl) / ciprofloxacin hydrochloride (CF).

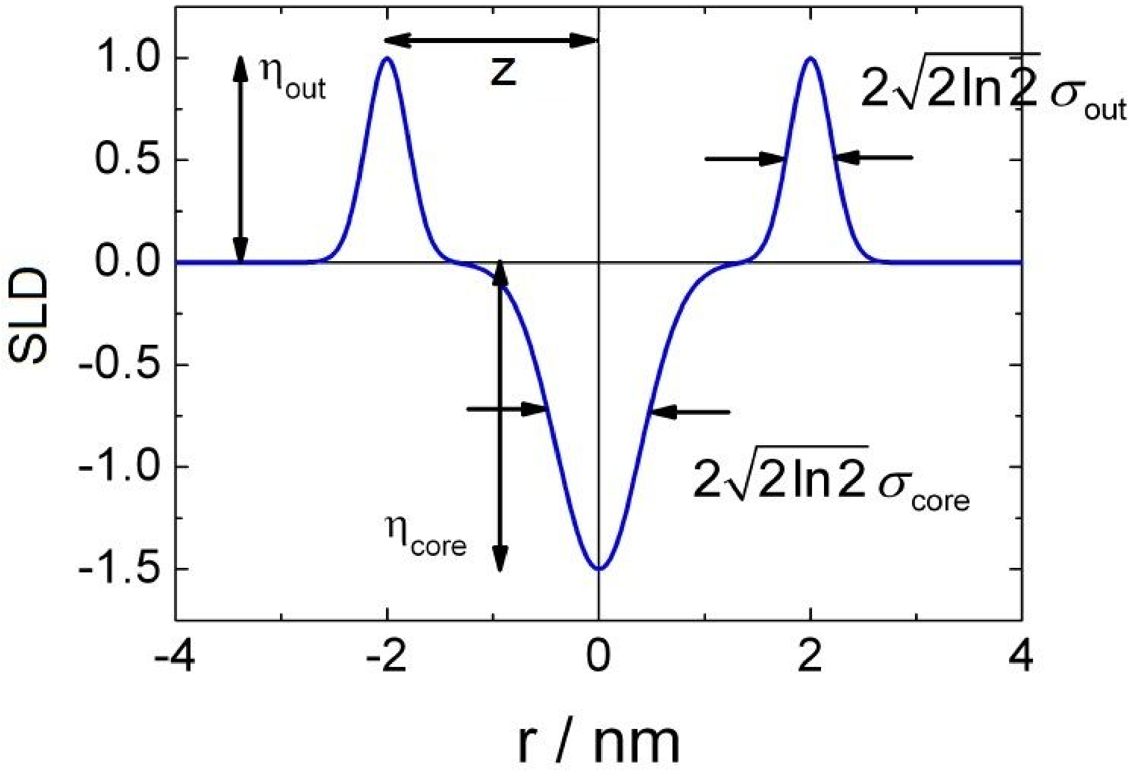

Figure S2. SLD profile for a lipid bilayer with a Gaussian electron density distribution.

Table S1. The composition (%w/v) of lipid nano-carrier formulations. PC – Soybean L-α-phosphatidylcholine; CHO – Cholesterol; DSPE-mPEG – 1,2-distearoyl-*sn*-glycero-3-phosphoethanolamine-N-[methoxy(polyethylene

glycol)] (ammonium salt) with different molecular weight; DSPE-PEG$_{2000}$-Mal – 1,2-distearoyl-*sn*-glycero-3-phosphoethanolamine-N-[maleimide(polyethylene glycol)-2000] (ammonium salt).

Liposome formulations	PC	CHO	DSPE-mPEG with different Mw	DSPE-PEG$_{2000}$-Mal
CL	0.773	0.077	-	-
LPEG	0.773	0.077	0.075	-
LPEG-Mal	0.773	0.077	-	0.075

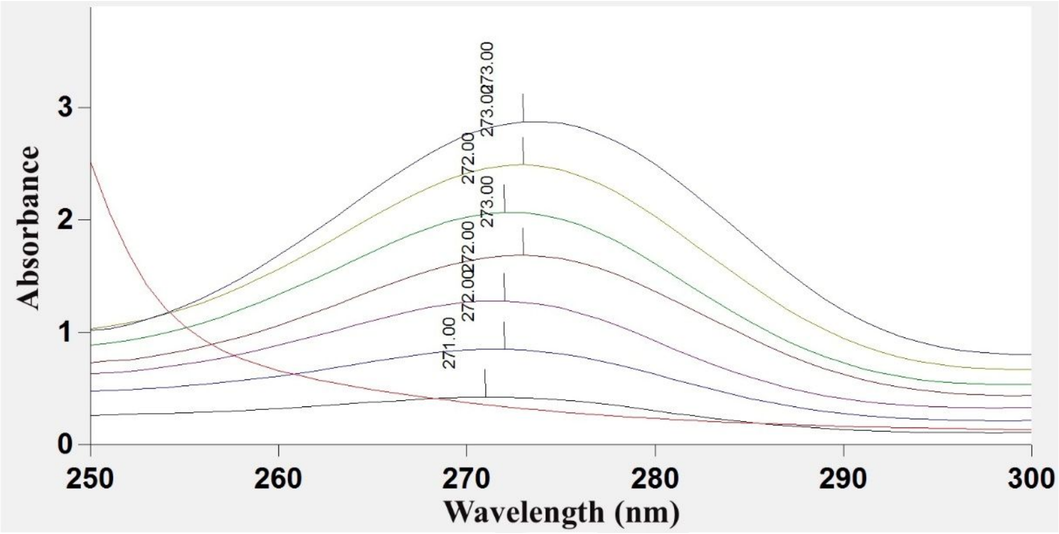

Figure S3. UV-Vis spectra with maximum absorbance peak for CF dissolved in 0.9% NaCl solution (Cary WinUV Scan Application software ver 4.20(470)).

Table S2. The encapsulation efficiency (EE%) and loading capacity (LC%) of CL, LPEG2000, and LPEG2000-Mal with encapsulated CF. No statistically significant difference detected between CL, LPEG2000, and LPEG2000-Mal.

Liposome formulations	EE%	LC%
CL	27 ± 13	3 ± 2
LPEG2000	28 ± 9	3 ± 1
LPEG2000-Mal	27 ± 13	3 ± 1

Table S3. The CF cumulative release (%) from CL, LPEG2000, and LPEG2000-Mal with encapsulated CF. No statistically significant difference detected between CL, LPEG2000, and LPEG2000-Mal (n = 5).

Time	CF release from liposomes (%)		
	CL	LPEG2000	LPEG2000-Mal
10 min	6.8 ± 0.5	6.5 ± 0.9	6.1 ± 0.8
20 min	14.7 ± 1.6	12.6 ± 1.1	14.6 ± 0.5
30 min	20.8 ± 0.9	19.1 ± 1.2	21.5 ± 1.0
1 hour	37.3 ± 2.6	33.4 ± 2.5	39.2 ± 1.9
2 hours	53.8 ± 4.3	50.4 ± 3.3	57.5 ± 3.3
4 hours	67.6 ± 4.8	70.8 ± 2.7	78.3 ± 4.5
8 hours	85.8 ± 3.4	85.4 ± 2.9	91.7 ± 5.2
12 hours	94.9 ± 2.6	94.9 ± 2.7	99.6 ± 5.3
18 hours	98.6 ± 3.0	100.5 ± 3.2	103.5 ± 6.7
24 hours	101.4 ± 2.8	103.0 ± 3.5	104.3 ± 5.8

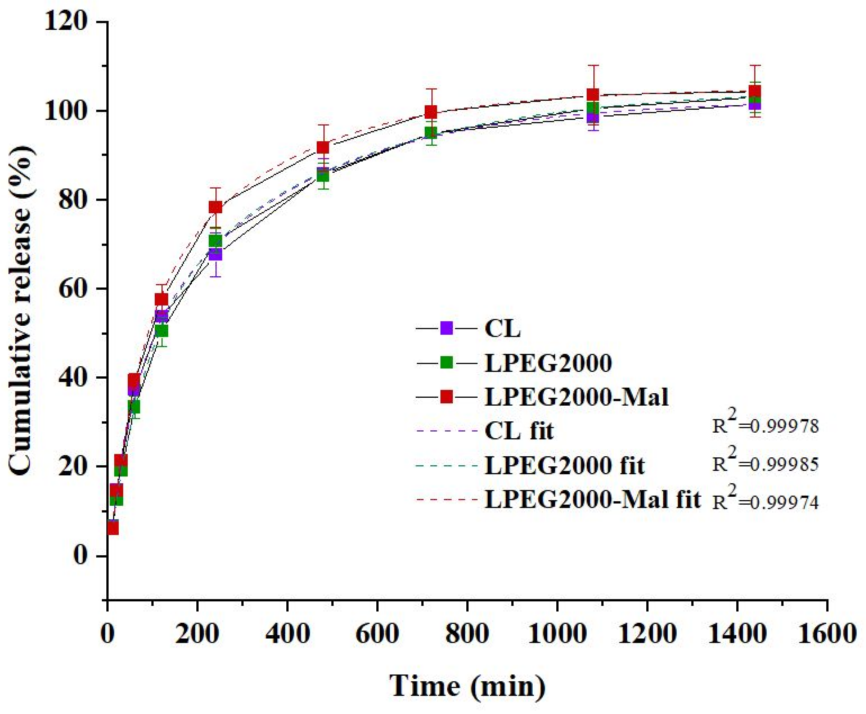

Figure S4. The selected release profiles of CF from CL, LPEG2000, LPEG2000-Mal with their fitting (OriginPro 9.8.0.200 software).

Table S4. Average fluorescence intensity (a.u.) and AUC values for the mucoadhesion test of 1 mg/mL FITC-dextran solution (negative control), CL, LPEG2000, and LPEG2000-Mal with entrapped 1 mg/mL NaFl on *ex vivo* bovine cornea (n = 3).

Time (min)	FITC-dextran	CL	LPEG2000	LPEG2000-Mal
	Average fluorescence values (a.u.)			
0	4.0990 ± 0.6477	22.2570 ± 3.5826	20.0086 ± 6.7985	24.6863 ± 1.3756
5	0.0217 ± 0.0116	1.5016 ± 0.9111	0.9423 ± 1.0134	0.4637 ± 0.2393
10	0.0060 ± 0.0061	0.2930 ± 0.1960	0.5516 ± 0.8143	0.1757 ± 0.1042
15	0.0004 ± 0.0001	0.2070 ± 0.1696	0.3466 ± 0.4960	0.0997 ± 0.0322
20	0.0021 ± 0.0025	0.2133 ± 0.1635	0.3060 ± 0.4356	0.0920 ± 0.0550
25	0.0015 ± 0.0022	0.2220 ± 0.1905	0.2936 ± 0.4186	0.1017 ± 0.0722
30	0.0015 ± 0.0022	0.2100 ± 0.2053	0.2853 ± 0.4171	0.1093 ± 0.1028
**Time (min)**	**Intensity (%)**			
0	100 ± 0	100 ± 0	100 ± 0	100 ± 0
5	0.5521 ± 0.3187	7.2622 ± 4.8262	3.8807 ± 3.7954	1.8466 ± 0.8716
10	0.1595 ± 0.1786	1.4249 ± 1.0186	2.2628 ± 3.0220	0.7282 ± 0.4748
15	0.0096 ± 0.0022	1.0057 ± 0.8750	1.4541 ± 1.8192	0.4048 ± 0.1323
20	0.0536 ± 0.0646	1.0393 ± 0.8430	1.2757 ± 1.5998	0.3685 ± 0.2046
25	0.0411 ± 0.0618	1.0843 ± 0.9738	1.2214 ± 1.5386	0.4063 ± 0.2710
30	0.0403 ± 0.0625	1.0296 ± 1.0399	1.1677 ± 1.5481	0.4335 ± 0.3890
**AUC**	254 ± 2	312 ± 44	303 ± 61	270 ± 6

Table S5. Average fluorescence intensity (a.u.) and AUC values for the mucoadhesion test of 1 mg/mL FITC-dextran solution (negative control), CL, LPEG2000, and LPEG2000-Mal with entrapped 1 mg/mL NaFl on *ex vivo* bovine conjunctiva (n = 3).

Time (min)	FITC-dextran	CL	LPEG2000	LPEG2000-Mal
	Average fluorescence values (a.u.)			
0	10.6157 ± 3.7292	27.7734 ± 6.6413	19.4320 ± 3.5161	29.8354 ± 9.6517
5	0.4167 ± 0.3403	5.2411 ± 1.1495	7.3466 ± 0.7682	23.4131 ± 10.8949
10	0.3801 ± 0.2644	4.4648 ± 1.1617	5.7646 ± 0.6547	22.4901 ± 12.5751

15	0.2994 ± 0.1910	4.1711 ± 1.0781	4.9870 ± 0.4073	19.4528 ± 11.2541
20	0.2554 ± 0.2308	4.2368 ± 1.1407	4.1743 ± 0.3948	18.5281 ± 9.9682
25	0.2247 ± 0.1906	4.0434 ± 1.1249	4.0036 ± 0.6481	18.1014 ± 9.7800
30	0.2091 ± 0.2151	3.9651 ± 1.1390	3.3330 ± 0.3581	16.0661 ± 8.6768
**Time (min)**	**Intensity (%)**			
0	100 ± 0	100 ± 0	100 ± 0	100 ± 0
5	3.5082 ± 1.9147	19.4055 ± 5.5685	39.0886 ± 11.1375	76.3710 ± 17.0051
10	3.3125 ± 1.1437	16.6429 ± 5.9773	30.7171 ± 9.1255	72.3537 ± 24.5654
15	2.6388 ± 0.7869	15.4950 ± 5.2615	26.5446 ± 7.4293	62.7429 ± 22.9016
20	2.1023 ± 1.3966	15.6066 ± 4.6756	22.2782 ± 6.6655	59.8132 ± 18.7892
25	1.8778 ± 1.0672	15.0201 ± 5.3500	21.5674 ± 7.9968	58.1160 ± 18.2375
30	1.6661 ± 1.3573	14.5890 ± 4.5216	17.8094 ± 5.4591	51.6241 ± 16.2074
**AUC**	321 ± 35	697 ± 145	996 ± 224	2026 ± 547
